# Access to Health Services in Older Minority Ethnic Groups with Dementia: A Systematic Review

**DOI:** 10.1111/jgs.16929

**Published:** 2020-11-24

**Authors:** Melissa Co, Elyse Couch, Qian Gao, Scarlett Mac‐Ginty, Jayati Das‐Munshi, Matthew Prina

**Affiliations:** ^1^ Department of Health Service and Population Research Institute of Psychiatry, Psychology and Neuroscience, King's College London London UK; ^2^ Department of Psychological Medicine Institute of Psychiatry, Psychology and Neuroscience, King's College London London UK; ^3^ South London and Maudsley NHS Trust London UK

**Keywords:** dementia, race/ethnicity, service use, hospital, memory services

## Abstract

**BACKGROUND/OBJECTIVES:**

While it is acknowledged that minority ethnic (ME) groups across international settings face barriers to accessing care for dementia, it is not clear whether ME groups access services less frequently as a result. The objective of this review is to examine whether ME groups have longer delays before accessing dementia/memory services, higher use of acute care and crisis services and lower use of routine care services based on existing literature. We also examined whether ME groups had higher dementia severity or lower cognition when presenting to memory services.

**DESIGN:**

Systematic review with narrative synthesis.

**SETTING:**

Nonresidential medical, psychiatric, memory, and emergency services.

**PARTICIPANTS:**

Twenty studies totaling 94,431 older adults with dementia or mild cognitive impairment.

**MEASUREMENTS:**

We searched Embase, Ovid MEDLINE, Global Health, and PsycINFO from inception to November 2018 for peer‐reviewed observational studies which quantified ethnic minority differences in nonresidential health service use in people with dementia. Narrative synthesis was used to analyze findings.

**RESULTS:**

Twenty studies were included, mostly from the U.S. (n = 13), as well as the UK (n = 4), Australia (n = 1), Belgium (n = 1), and the Netherlands (n = 1). There was little evidence that ME groups in any country accessed routine care at different rates than comparison groups, although studies may have been underpowered. There was strong evidence that African American/Black groups had higher use of hospital inpatient services versus U.S. comparison groups. Primary care and emergency services were less well studied. Study quality was mixed, and there was a large amount of variability in the way ethnicity and service use outcomes were ascertained and defined.

**CONCLUSION:**

There is evidence that some ME groups, such as Black/African American groups in the U.S., may use more acute care services than comparison populations, but less evidence for differences in routine care use. Research is sparse, especially outside the U.S.

## INTRODUCTION

### Background

Despite evidence that minority ethnic (ME) older adults in the U.S. and UK have higher risks of dementia,^1^, ^2^, ^3^, ^4^ it is widely reported they face numerous barriers to accessing dementia care. These include stigma, discrimination, racism, language barriers, different conceptualizations of dementia etiology, and negative experiences navigating health services.^5^, ^6^, ^7^, ^8^ Interviews with African‐Caribbean and South Asian individuals in the UK found that dementia and related services were perceived as phenomena of other cultures, rather than their own.^9^, ^10^ However, barriers have also been reported for comparison populations. In the UK, both Black Caribbean and White British individuals with dementia used stigmatizing language when describing dementia,^11^ and the misconception that dementia is a normal part of aging has been reported across British Black Caribbean, British South Asian, and White British groups.^6^, ^9^, ^11^

These barriers may result in disparities in health service use. A 2002 review reported that ME groups in the UK underutilized services, citing barriers to access.^12^ Another review in 2010 found that ME groups in the U.S. had higher levels of cognitive impairment upon presentation to dementia services versus comparison populations.^13^ This supports qualitative findings from the U.S. and UK that some ME families delay help‐seeking until a “crisis point” is reached and hospital or crisis services are needed.^7^, ^14^, ^15^ ME groups might use more acute and emergency services rather than accessing recommended primary and outpatient secondary care pathways^7^, ^16^, ^17^ and might have longer delays between onset of symptoms and presentation to dementia/memory services. Frequent and early use of primary care and outpatient services may result in better dementia management, whereas frequent use of emergency or inpatient care may indicate inconsistent management. However, few studies have quantified differences in the rates at which ME groups present to services.

This is also important from an economic perspective, as emergency and inpatient services incur higher costs to payors (including patients themselves). In the U.S., a 2010 study found that non‐Hispanic White persons with dementia had higher outpatient care costs, but not higher inpatient costs, as compared with ME groups.^18^ Costs can also differ between ME groups—a 2012 study found that non‐Hispanic Black patients with Alzheimer's disease (AD) had higher care costs than Hispanic patients.^19^ Service use disparities additionally affect out‐of‐pocket expenses, potentially widening existing socioeconomic disparities. In one study, out‐of‐pocket health expenses for Black individuals with dementia added up to 84% of their household wealth as compared with 32% in non‐Black groups.^20^

Much of our knowledge of ME groups' pathways to care thus far has come from qualitative studies.^6^ However, quantitative studies comparing rates of service use/access will help inform whether initiatives to improve access should focus on specific groups or the general population. The objective of this review is to determine whether ME groups have longer delays before accessing dementia/memory services, higher use of acute/crisis services (emergency department attendances and inpatient admissions), and lower use of routine services (outpatient and primary care services). As a secondary objective, we examine whether ME groups present to services with greater cognitive impairment or dementia severity, as this is thought to indicate longer delays to access. We expect to find similar service use patterns across countries because similar barriers have been reported for ME groups generally, but due to different health systems and racial/ethnic experiences, contexts, and histories, we consider results from different countries separately.

## METHODS

A protocol for this review was registered in PROSPERO (CRD42018118132) (Supplementary Appendix S1). The PRISMA checklist is reported in Supplementary Appendix S2.

### Eligibility Criteria

To be included, studies needed to fulfill these criteria: (1) observational design, (2) reported quantitative results on differences in health service use or access by participant ethnicity, and (3) participants had dementia (any) or mild cognitive impairment. We defined health services to include any inpatient admission or outpatient visit with general medical care, psychiatric services, memory clinics, or emergency services. Residential and social care services, such as nursing home and hospice, were excluded; these services include additional nonmedical components, may be less comparable to other health services, and have been reviewed previously.^13^, ^21^, ^22^ As a secondary objective, we included studies measuring cognitive impairment or dementia severity at presentation to services. These were captured by our search strategy, but because we did not initially include these in the protocol, their results are discussed separately.

We excluded studies investigating: (1) services specific to unrelated conditions, (2) diagnosis rates, (3) advanced care directives or intentions/attitudes toward services, and (4) economic costs, if service use was not separately reported.^23^

Further detail on including ethnicity is in Supplementary Box S1.

### Search

We searched Embase, Ovid MEDLINE, Global Health, and PsycINFO from inception to November 7, 2018 for peer‐reviewed journal articles fitting our inclusion/exclusion criteria. The search (Supplementary Appendix S3) followed this structure: (dementia OR Alzheimer's disease) AND (ethnicity OR race) AND (service use), including subject headings and synonyms of included terms.

### Study Selection

Titles and abstracts of articles were screened in full by one reviewer (MC). Two reviewers (EC, QG) additionally screened a 30% sample of the records using Rayyan.^24^ Full texts of abstracts included by any author at this stage were then reviewed by one reviewer (MC) for inclusion using EndNote, and a random 10% were checked by a second reviewer (SM‐G). A third reviewer (MP) was consulted in the case of any disagreements.

### Data Extraction

Data was extracted from included articles by one author (MC) using Excel. Information extracted included: study name, country, setting, type of service, year conducted, population, ethnicity groups (and definitions), sample size, proportion of female participants, mean age, dementia type, outcome definition, type of analysis, crude and adjusted statistics, confidence intervals (CIs), covariates, and key findings.

### Risk of Bias in Individual Studies

Risk of bias was assessed using the Newcastle‐Ottawa scale (NOS),^25^ with two added questions capturing: (1) whether ethnicity was the primary predictor variable (yes, no, exploratory) and (2) whether ethnic differences were discussed explicitly (rather than just reported in tables). Two NOS versions were used: one modified for cross‐sectional studies,^26^ and the NOS for cohort studies. Studies were rated on each outcome included, and star ratings were averaged if ratings differed between outcomes.

For ascertainment of exposure, studies were given one (out of one) star if they stated that ethnicity was self‐identified.^23^ This adapts the cross‐sectional version to have the same stars per category as the cohort scale. Studies using routine data were assumed not to have nonresponse or loss‐to‐follow‐up but are noted. Samples were considered unrepresentative if they drew from populations thought to differ in ME group representation (e.g., nursing homes).^13^

### Synthesis of Results

We used narrative synthesis to summarize findings.^27^ Study characteristics and results were tabulated. Studies were grouped by country, service, and ME groups studied to compare patterns. Because ME groups vary between countries, we did not compare results across countries.

Although it was necessary to group overlapping ethnicity categories to synthesize results, we adopt the terminology used by study authors when discussing individual studies. For example, “Black” ethnicity groups in the U.S. generally overlap with “African American” ethnicity groups and are combined in our synthesis, but when individual studies are mentioned the study authors' term is used. Some individuals might identify with one term but not the other. We discuss our use of ethnicity terms in further detail in Supplementary Box S1.

## RESULTS

Of 8,977 records obtained, 8,846 were excluded after screening titles and abstracts, and full texts of 131 were reviewed for eligibility. Twenty full‐text articles were included in narrative synthesis.

**Figure 1:**
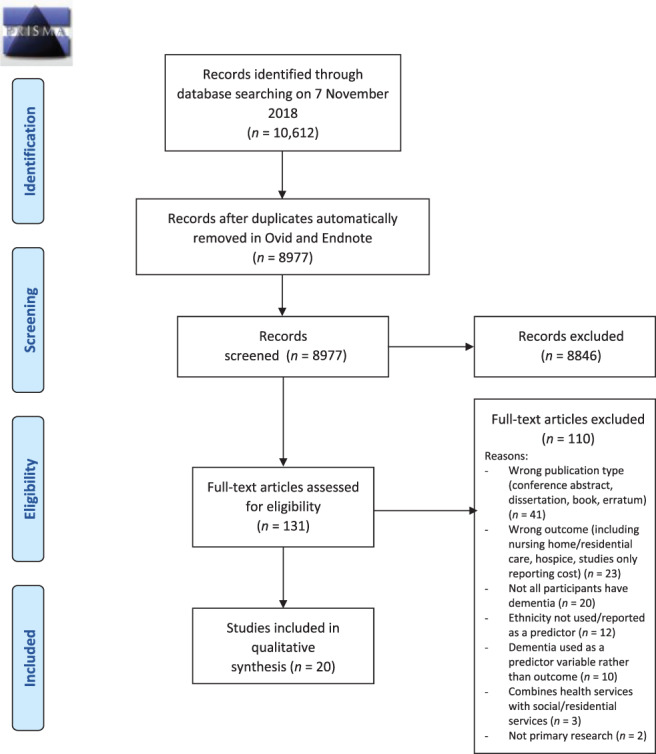
PRISMA flow diagram. This figure depicts the process used to identify and screen articles found in the search, including numbers of papers excluded and exclusion reasons for full‐text articles.

Most excluded articles were conference abstracts and non–peer‐reviewed publications (n = 41). The full selection process is presented in Figure 1.

### Study Characteristics

Supplementary Table S1 summarizes the number of studies found in each country for each race/ethnicity group and service. Thirteen studies were from the U.S., four from the UK, and one each from Australia, Belgium, and the Netherlands. Study sizes ranged from 86 to 55,827 participants, totaling 94,431 participants altogether. Services included: primary care (n = 1), memory services (memory clinics, Alzheimer's Disease Centers, and memory assessment centers) (n = 8), general outpatient services (n = 2), inpatient and intensive care unit (ICU) (n = 10), and emergency services (n = 1). Study characteristics can be found in Table 1, with detailed demographics in Supplementary Table S2.

**Table 1:** Study Characteristics

Study	Services evaluated	Population	Ethnicities studied (study authors' terminology)
Australia			
LoGiudice et al (2001)^28^	Memory clinic	Patients from NorthWest hospital memory clinic	English‐speaking background (ESB); non‐English‐speaking background (NESB)
Belgium			
Segers et al (2013)^29^	Memory clinic	Newly referred patients at the Memory Clinic of the Brugmann University Hospital in Brussels with pure AD or AD with vascular lesions (subset of full sample)	Belgian‐born; European immigrants; non‐European immigrants
The Netherlands			
Agyemang et al (2017)^30^	Hospital	Patients with first hospitalization or first day clinic attendance for dementia from data linkage of Dutch national registers	Dutch; Indonesian; Turkish; Surinamese; Antillean (based on place of birth)
United Kingdom			
Knapp et al (2016)^31^	Hospital (general and mental health)	Individuals with a diagnosis of Alzheimer's Disease in South London and Maudsley NHS Foundation Trust and not initially living in a care home	(1) Caribbean, African, or other Black; (2) East Asian or South Asian; (3) Mixed, unknown, and other; (4) White British or Other White
Park et al (2017)^32^	Memory Assessment Services	Patients with a first referral to a memory assessment service with suspected dementia	White/White British; Other ethnicity
Sleeman et al (2018)^33^	Emergency department	Patients 60+ with a diagnosis of dementia in South London and Maudsley NHS Trust who died after 2008	White British; Other White; African Caribbean; Other; Not known
Tuerk and Sauer (2015)^34^	Memory clinic	Patients referred, assessed, and diagnosed with dementia at the Southwark and Lambeth Memory Service (only included subset diagnosed with dementia)	White British; Black and minority ethnic (BME, including only Caribbean and African)
U.S.			
Akpaffiong et al (1999)^35^	Psychiatric inpatient unit	Veterans Affairs Medical center patients with dementia (no delirium) who are admitted for behavioral disturbance	Caucasian; African American
Chow et al (2000)^36^	Primary care, Alzheimer's Disease Centre (ADC)	Patients evaluated at an Alzheimer's Disease Diagnosis and Treatment Centre in California	Caucasian; Filipino; Asian (includes Chinese, Japanese, Korean)
Cohen and Carlin (1993)^37^	Dementia assessment center	Consecutive patients from Brooklyn Alzheimer's Disease Assistance Centre	White; Black
Cox (1996)^38^	Hospital	Purposive sample of patients admitted from home, with no terminal illness, living with their carer, hospitalized in five acute care hospitals in and around Washington, D.C., and referred by hospital social workers to the study	African American; White
Gaugler et al (2006)^39^	Hospital	Participants of the Medicare Alzheimer's Disease Demonstration Evaluation (MADDE) diagnosed with dementia and living at home in MADDE catchment areas across the U.S. (NY, IL, IN, OR, OH, WV, MN, FL)	Latino; Caucasian; African American
Gessert et al (2006)^40^	Hospital, ICU	Nursing home residents in Minnesota and Texas over 67 years of age in Medicare Minimum Data Set	White; non‐White
Husaini et al (2003)^41^	Hospital	Medicare beneficiaries 65+ years old who were discharged in 2008, from the Tennessee Hospital Discharge database	White; Black
Husaini et al (2015)^42^	Hospital, outpatient visits, physician visits, emergency services	5% random sample of Medicare beneficiaries who filed claims for services	White; African American
Livney et al (2011)^43^	Alzheimer's Disease Centre	Patients 50+ years of age at the University of Pennsylvania AD Centre with a primary consensus diagnosis of AD	African American; Hispanic/Latino; White non‐Hispanic
Miller et al (2009)^44^	Hospital, outpatient (including AD‐related, mental health, medical‐surgical)	Participants from the Clinical Antipsychotic Trial of Intervention Effectiveness‐AD trial who: have AD or probable AD, live at home or in assisted living, have had severe delusions, hallucinations, aggression, or agitation	Non‐Hispanic White; Other
Ornstein et al (2018)^45^	Hospital, ICU	Participants of the Washington Heights‐Inwood Columbia Aging Project study: Medicare beneficiaries 65+ in northern Manhattan. Results are reported here from the subgroup of participants who were followed until death	Non‐Hispanic White; non‐Hispanic Black; Hispanic
Watari and Gatz (2004)^46^	Alzheimer's Disease Centre, services used prior to attendance	Community‐residing patients of the St. Barnabas AD Diagnostic and Treatment Centre	Korean American; African American; Latino/a; European American
Weiner et al (2003)^47^	Alzheimer's Disease Centre	Patients with probable and possible AD from University of Texas Southwestern Alzheimer's Disease Centre and outreach clinics in Oklahoma and Texas	Native American

Abbreviations: AD, Alzheimer's disease; ICU, intensive care unit; NHS, National Health Service.

In the U.S., all studies included White, European American, Caucasian, or non‐Hispanic White groups. Nine studies included African American, Black, or non‐Hispanic Black groups. Four studies included Hispanic or Latino groups. Two studies included groups that usually fall under Asian American ethnicity categories (Filipino American, Asian American, and Korean American).^36^, ^46^ Only one study included a Native American group.^47^

In the UK, all four studies included a White or White British group. Three studies included Black Caribbean/Black African/other Black groups. Only one study included a separate other White group,^33^ and only one included East Asian/South Asian and mixed/unknown/other groups.^31^

The study from Australia classified ethnicity as those from English‐speaking backgrounds (ESB) and non‐English‐speaking backgrounds (NESB).^28^ In the Netherlands and Belgium, ethnicity was defined by country of birth/migration.^29^, ^30^

### Risk of Bias within Studies

Results from NOS assessments are in Table 2 and Supplementary Tables S3 and S4. Study quality was mixed. Cross‐sectional studies tended to score lower than cohort studies because service use outcomes were often not the primary outcome of interest but rather reported in baseline demographics. Thus, sample size was not tailored to these outcomes, statistical tests not always reported, and effects not adjusted for other variables. Poor reporting of how ethnicity was defined or ascertained made it difficult to compare across studies. Only five studies (all from the U.S.) ascertained ethnicity based on self‐report.^36^, ^37^, ^43^, ^45^, ^47^ In most studies, ethnicity was assigned based on records, which may have been filled out by someone other than the individual themselves and may not be accurate.

**Table 2:** Newcastle Ottawa Scale Results

	Selection (out of 4)	Comparability (out of 2)	Outcome (out of 3)	Ethnicity as the main predictor variable (yes or no)	Ethnicity reported in results (yes or no)
**Cohort**					
Agyemang et al (2017)	***	**	***	yes	yes
Chow et al (2000)	****		*	yes	yes
Knapp et al (2016)	***	**	**	no	yes
Miller et al (2009)	*	**		no	yes
Ornstein et al (2018)	****		**	yes	yes
Sleeman et al (2018)	**	**	***	no	yes
**Cross‐sectional**					
Akpaffiong et al (1999)	*		**	yes	yes
Chow et al (2000)	***		**	yes	yes
Cohen and Carlin (1993)	***			yes	yes
Cox (1996)			*	yes	yes
Gaugler et al (2006)			*	yes	yes
Gessert et al (2006)	*	*	***	no	yes
Husaini et al (2003)	*		**	yes	yes
Husaini et al (2015)	**		**	yes	yes
Livney et al (2011)	**	**	*	yes	yes
LoGiudice et al (2001)	**		**	yes	yes
Park et al (2017)	*	**	***	no	yes
Segers et al (2013)	**	*	**	yes	yes
Tuerk and Sauer (2015)	**		***	yes	yes
Watari and Gatz (2004)	***		*½	yes	yes
Weiner et al (2003)	***		*	yes	yes

*Note*: Asterisks denote the number of stars awarded in each category for the quality assessment rating.

### Results of Individual Studies

Key study findings can be found in Tables 3, 4, and Supplementary Table S8, and full results in Supplementary Tables S5, S6, and S7.

**Table 3:** Key Findings on Service Use

Study	Country	Ethnicities studied	Key findings
Segers et al (2013)	Belgium	Belgian‐born, European immigrants, non‐European immigrants	Delay to presentation to memory clinic: no difference
Agyemang et al (2017)	Netherlands	Dutch, Indonesian, Turkish, Surinamese, Antillean	Readmission risk: no difference after adjustment
Knapp et al (2016)	UK	(1) Caribbean, African, or other Black, (2) East Asian or South Asian, (3) Mixed, unknown, and other, (4) White British or Other White	Mental health inpatient admissions over 6‐month period: no difference General inpatient admissions over 6‐month periods: lower probability in Caribbean/African group and East/South Asian group compared with White/White British group
Sleeman et al (2018)	UK	White British, Other White, African Caribbean, other, not known	Emergency department attendance in last year of life: no difference
Chow et al (2000)	U.S.	Caucasian, Filipino, Pacific Islander (not included in statistical analyses), Asian	Frequency of use of primary medical care prior to baseline: no difference Frequency of referrals to primary medical care: no difference Frequency of referrals completed to primary medical care: no difference
Cohen and Carlin (1993)	U.S.	White, Black	Years of having symptoms prior to presenting to assessment center: no difference Having a prior evaluation before presenting to center: no difference
Cox (1996)	U.S.	African American, White	Number of hospitalizations in last 12 months: Higher in African American group compared with White group Number of days spent in hospital in last 12 months: no difference
Gaugler et al (2006)	U.S.	Latino, Caucasian, African American	Number of hospital stays in past 6 months: Higher in African American group as compared with Caucasian group
Gessert et al (2006)	U.S.	White, non‐White	Use of hospital in last 90 days of life: Higher in non‐White group compared with White group Use of ICU in last 90 days of life: Higher in non‐White group compared with White group
Husaini et al (2003)	U.S.	White, Black	Additional admissions to hospital: Higher in Black ethnicity groups compared with White ethnicity groups Number of hospital days: Higher in Black ethnicity groups compared with White ethnicity groups
Husaini et al (2015)	U.S.	White, African American	Average number of inpatient days: Higher in African American group compared with White group Average number of outpatient visits: no difference Average number of physician visits: no difference% of group who had visited emergency services: no difference
Livney et al (2011)	U.S.	African American, Hispanic/Latino, White non‐Hispanic	Time from onset to initial presentation to AD center: Lower in African American group compared with White non‐Hispanic group, but no other differences
Miller et al (2009)	U.S.	Non‐Hispanic White, Other	Inpatient hospital use: no difference after adjustment for sociodemographic factors, cognition (MMSE), ADL, quality of life, etc. Any outpatient use: Higher in non‐Hispanic White group as compared with other ethnicity groups AD‐related outpatient use: Higher in non‐Hispanic White group as compared with other ethnicity groups Mental‐health outpatient use: no difference after adjustment for sociodemographic factors, cognition (MMSE), ADL, quality of life, etc. Medical‐surgical outpatient use: no difference after adjustment sociodemographic factors, cognition (MMSE), ADL, quality of life, etc.
Ornstein et al (2018)	U.S.	non‐Hispanic White, non‐Hispanic Black, Hispanic	Number of hospital admissions (from diagnosis to death): Higher in non‐Hispanic Black group as compared with non‐Hispanic White group, no difference between Hispanic and non‐Hispanic White group Number of hospital days: Higher in non‐Hispanic Black group as compared with non‐Hispanic White group, no difference between Hispanic and non‐Hispanic White group Number of ICU days: no difference
Watari and Gatz (2004)	U.S.	Korean American, African American, Hispanic/Latino, White/European American	Years to seeking help at AD center: no statistical difference reported, but higher years to seek help in Latino American group compared with Korean American group when calculated using data presented. Mean number of services used prior to attendance: no difference
Weiner et al (2003)	U.S.	Native American, White	Time to evaluation from onset: no difference

Abbreviations: AD, Alzheimer's disease; ADL, activities of daily living; ICU, intensive care unit; MMSE, mini‐mental state examination.

**Table 4:** Key Findings on Severity/Cognition at Presentation

Study	Country	Ethnicities studied	Severity/cognition at presentation
Logiudice et al (2001)	Australia	English‐speaking background (ESB), non‐English‐speaking background (NESB)	MMSE, CDR, CAMCOG at presentation to memory clinic: People of non‐English‐speaking backgrounds had lower scores at presentation.
Segers et al (2013)	Belgium	Belgian‐born, European immigrants, non‐European immigrants	MMSE: lower in non‐European immigrant group compared with European immigrant and Belgian‐born groups.
Park et al (2017)	UK	White/White British, Other ethnicity	Cognitive function at presentation to memory assessment services: Higher in White/White British group as compared with “Other” group of individuals not identified as White/White British
Tuerk and Sauer (2015)	UK	White British, Black and minority ethnic (BME, including only Caribbean and African backgrounds because of small numbers of other ethnicities)	ACE‐R at first presentation to memory service: lower in Black Caribbean/Black African group as compared with White British group MMSE at first presentation to memory service: no differences found
Akpaffiong et al (1999)	U.S.	Caucasian, African American	MMSE score at admission to psychiatric inpatient for behavioral disturbances: no difference
Chow et al (2000)	U.S.	Caucasian, Filipino, Pacific Islander (not included in statistical analyses), Asian	MMSE at presentation: Lower mean MMSE at baseline for Filipino and Asian groups compared with Caucasian group
Livney et al (2011)	U.S.	African American, Hispanic/Latino, White non‐Hispanic	Cognition (MMSE, Global Cognition Index) at presentation: Lower in African American group compared with White non‐Hispanic even after controlling for education. Severity (DSRS, CDR) at presentation: Higher in the African American group as compared with the White non‐Hispanic group, higher in the Latino group as compared with the African American group for CDR. Differences between Latino and White non‐Hispanic groups were attenuated after adjusting for education.
Watari and Gatz (2004)	U.S.	Korean American, African American, Hispanic/Latino, White/European American	MMSE at presentation: lower in African American group compared with European American group Severity at presentation based on BDRS‐CERAD: no difference
Weiner et al (2003)	U.S.	Native American, White	MMSE at evaluation at AD center: no difference

Abbreviations: ACE‐R, Addenbrooke's Cognitive Examination Revised; AD, Alzheimer's disease; BDRS‐CERAD, Blessed–Roth Dementia Scale Rating (Consortium to Establish a Registry for Alzheimer's Disease version); CAMCOG, cognitive section of the Cambridge Examination for Mental Disorders of the Elderly; CDR, clinical dementia rating; DSRS, Dementia Severity Rating Scale; MMSE, mini‐mental state examination.

### Service Use in the U.S.

There was very little evidence that African American/Black groups accessed routine care services at different rates compared with other ethnic groups. Two out of three studies suggested that there were no differences in delay to accessing memory services from onset of dementia, although African American individuals had slightly shorter mean time from onset to presentation (3.1, standard deviation (SD) = 2.12) compared with White non‐Hispanic individuals (3.4, SD = 2.29, *P* = .05) but not Latino individuals (3.3, SD = 3.32, *P* = .30) in one study.^43^ Another study found no difference in use of outpatient services or physician visits.^42^

However, in studies on inpatient hospital use (n = 5), evidence indicated that African American/Black groups had higher rates of inpatient service use compared with reference groups (White/non‐Hispanic White/Caucasian). All studies found either increased time spent in hospital or greater number of admissions, although one study found no difference in days spent in ICU.^45^ There was no difference in use of emergency services compared with White groups in one study.^42^

For the secondary objective, evidence indicated that African American/Black groups had lower cognition scores (3.1^43^ and 5.3^46^ points lower on the mini‐mental state examination (MMSE)) at presentation to memory services (n = 2) but not at admission to a psychiatric inpatient unit (n = 1).

Of the studies which included Hispanic or Latino groups (n = 4), none reported differences in use of either routine care services or acute care services, specifically, no evidence of difference in time to presentation to AD center (n = 2) or number of hospital stays and inpatient/ICU days (n = 2).

Of the two studies measuring severity/cognition at presentation to memory services, one found no difference^46^ and one found higher severity and/or lower cognition scores in the Latino group (mean MMSE 15.1, SD = 5.91) as compared with both African American (17.6, SD = 5.16, *P* = .64) and non‐Hispanic White groups (20.7, SD = 5.34, *P* = .0074), although differences were attenuated after controlling for education.^43^

One paper found no difference in Native American groups' time to accessing memory services (*P* = .39), although it should also be noted that these services included outreach clinics specifically targeted toward Native American individuals with dementia.^47^ There was also no difference in cognition scores at presentation to the memory service (*P* = .33).

Two studies included Asian American groups in routine care settings. One found no differences between Korean Americans and other ethnic groups studied in years to seeking help at an AD center (mean = 3.27, SD = 2.68 in Korean American group) or mean number of services used prior to attendance (after controlling for education and income).^46^ The other found no differences in referrals or use of primary care among Filipino Americans and Asian Americans compared with Caucasian Americans, either before or after memory service attendance, and 83–100% in all groups had accessed primary care services.^36^

However, Asian American and Filipino American groups had lower mean MMSE scores at presentation to memory services in this study (Asian: 15.4, SD = 7.1, Filipino: 15.1, SD = 7.6, Caucasian 17.7, SD = 7.3, *P* < .01).^36^ No difference in cognition/severity was found for the Korean American group.^46^

Two papers compared White/non‐Hispanic White groups with non‐White/other ethnicity groups. Compared with other groups in one study, White patients had higher odds of using both general and AD‐specific outpatient services per month, but similar odds of having an inpatient stay (general: odds ratio (OR) = 1.61, 95% CI = 1.10–2.52; AD‐specific: 1.53, 1.00–2.35; inpatient: 0.81, 0.43–1.51).^44^ However, the other study found that they were less likely to have inpatient or ICU stays at the end of life (odds of hospitalization 1.54 and 2.41 times higher for the non‐White group in rural and urban nursing homes, respectively).^40^ However, definitions of “White” and “non‐White” groups may differ in these studies, as only one specified non‐Hispanic White ethnicity.

### Service Use in Australia, Belgium, the Netherlands, and the UK

None of the UK studies examined routine services. One study found that African Caribbean individuals with dementia had higher rates of emergency department attendance in the last year of life as compared with White/White British individuals.^33^ However, this was no longer evident after controlling for demographic and clinical factors (Incidence Rate Ratio 1.07, 95% CI = 0.95–1.19, *P* = .26), and there were no differences between other ME groups (overall mean = 2.1, SD = 2.3). Another study found lower risk of general hospital inpatient admissions in Caribbean/African groups (OR = 0.68, 95% CI = 0.53–0.88, *P* < .01) and East/South Asian groups (0.43, 95% CI = 0.25–0.73, *P* < .01) as compared with White British, although no difference was found for mental health admissions.^31^

Two UK studies examined cognition at presentation to memory services. One found that Black Caribbean/Black African groups had lower average scores on the Addenbrooke's Cognitive Examination‐Revised (48.7, SD = 11.2) compared with White British groups (57.4, SD = 13.5) but not the MMSE (mean = 20.1, SD = 4.1 vs 21.0, SD = 4.6) .^34^ The other found higher average cognitive scores in White/White British individuals as compared with individuals identifying with other ethnicity groups (OR = 1.3, 95% CI = 1.1–1.7).^32^

In the study from Belgium, there was no evidence for longer average time (years) to memory service presentation from first symptoms in either European (1.5, SD = 0.8) or non‐European (3.2, SD = 3.1) immigrant groups compared with the Belgian‐born group (1.9, SD = 1.8).^29^ However, the non‐European immigrant group had lower mean MMSE scores at presentation (14.0, SD = 6.4) compared with both the European immigrant group (19.5, SD = 6.2) and Belgian‐born group (22.2, SD = 4.6).

The study from the Netherlands found no difference in time to hospital readmission between Dutch, Surinamese, Turkish, Antillean, and Indonesian ethnic groups after controlling for age, sex, and comorbidities.^30^

In the study from Australia, NESB groups had lower MMSE (mean = 14.7, SD = 6.2, *P* < .001), cognitive section of the Cambridge Examination for Mental Disorders of the Elderly (CAMCOG) (49.2, SD = 20.8, *P* < .001), and clinical dementia rating (*χ* ^2^ = 14.3, *P* = .003) scores at presentation to memory clinic as compared with ESB groups (MMSE: 18.0, SD = 5.3; CAMCOG: 58.2, SD = 17.0).^28^

### Additional Analyses

Larger studies (>1,000 participants, n = 8 across all countries) tended to report differences in service use between ME groups and comparison groups on at least one outcome, with only one reporting no difference.^30^ In the U.S., the four studies finding no service use differences in any ethnic group were older (data from 1992 to 2002), although other older studies did report differences.^38^, ^39^, ^41^

Study quality may be associated with whether differences were reported; studies with at least 5/9 stars on the NOS more frequently found differences in both service use and severity/cognition, but there were also many exceptions.

## DISCUSSION

### Summary of Evidence

In this review, we sought to assess whether ME older adults with dementia were less likely to use routine care services and more likely to use acute and crisis services versus comparison populations. Although we did not limit our search by country, most studies were from the U.S., and we found few studies from other countries.

We found little evidence that ME groups in any country studied used fewer routine care services or reported longer delays to accessing memory services. However, few studies examined primary care services, so we cannot make conclusions about use of primary care.

There was strong evidence from the U.S. that African American/Black ethnic groups were more frequently admitted to hospitals as compared with reference groups. This finding is consistent with a large body of literature which has found higher rates of preventable hospitalizations for ambulatory care‐sensitive conditions in Black/African American versus non‐Hispanic White groups. Disparities have been found in populations of older adults and individuals with chronic conditions (including hypertension and diabetes, both associated with dementia) and have remained even after adjusting for socioeconomic factors and disease prevalence.^48^, ^49^, ^50^ These studies posited that lack of accessible outpatient care, poor quality of routine care support/disease management, different help‐seeking preferences, and discriminatory treatment of Black/African American groups in health services may underlie these hospitalization patterns,^48^, ^49^, ^50^ although our review found less evidence for disparities in routine dementia services. Because studies in our review did not indicate reasons for hospitalizations, it is unclear whether the increased rates of hospitalizations of Black/African American groups in our review were due to preventable hospitalizations for chronic physical conditions, a similar pattern of preventable hospitalizations for dementia, or other factors. Two of the four studies measuring inpatient stays in Black/African American groups also reported rates of comorbidities, both finding that rates of hypertension and diabetes were higher in the Black older adults in their samples versus comparison groups.^37^, ^42^ It is possible that both higher prevalence of conditions in study participants and higher risk of preventable hospitalizations for these conditions doubly contributed to the increased rates of hospitalization observed.

Higher rates of preventable hospitalizations have also been reported for Hispanic/Latino populations,^48^, ^49^, ^50^ but we found limited evidence for this in dementia.

Additional studies on emergency attendances are needed to clarify whether ME groups are more likely to present during crises, particularly if emergency care is their first contact with services.

Previous literature has pointed to lower cognitive scores at presentation as an indication that ME groups may delay access to care.^13^ We found evidence from the U.S., UK, Belgium, and Australia indicating that multiple ME groups had lower cognitive/severity scores at presentation to memory services. Although a few studies found no difference in cognition/severity scores at presentation, no studies found higher cognition/severity scores.

As we did not find differences in reported time delays to accessing memory services, it is possible that these cognition/severity differences are instead indicative of psychometric properties of tests. One study found that MMSE score differences were attenuated after controlling for education,^43^ which has been associated with MMSE.^51^, ^52^ Additionally, MMSE and other cognitive tests can be less sensitive for diagnosing dementia in certain ME groups, including Black American older adults,^53^, ^54^ although studies disagree as to how much this is explained by education.^55^ It is risky to assume that lower scores necessarily indicate delayed contact with services without measuring time to presentation.

The studies included were heterogenous in design, quality, ethnicity groups studied, and outcomes used. They also spanned many years, with the oldest data collected in 1989. How ethnicity is measured and how individuals identify may have changed over time,^56^ as well as trends in service use.

It is possible that studies not reporting differences were underpowered, as we found that higher quality and larger studies tended to report differences between groups. Few studies included power calculations, especially when service use was not the primary outcome. Thus, we are cautious of interpreting these results as evidence that service use is similar across groups.

Studies which adjusted for other factors suggest that differences might be partially explained by sociodemographic and clinical factors.^30^, ^33^, ^44^ Socioeconomic status particularly may confound the association between ethnicity and service use, especially where high out‐of‐pocket costs pose a barrier to accessing healthcare, such as in the U.S. Most studies did not account for this. Examining the intersection of other sociodemographic factors will provide a more nuanced understanding of service use disparities, as well as where to focus initiatives for improving access to routine care. In order to assess and improve healthcare accessibility for dementia for all groups, further research/reviews should examine racial/ethnic differences in possible drivers of service use disparities, including out‐of‐pocket expenses, patient experiences, and preventable hospitalizations.

### Strengths and Limitations

We included many search terms related to ethnicity and service use to be as comprehensive as possible. We also did not exclude based on article language. However, we may have unknowingly omitted terms used to describe race/ethnicity in other countries of which we were unaware. We may have also missed studies which did not include ethnicity terms in their title, abstract, or subject headings.

We excluded residential and social care services from our review, but these services also provide medical care which may impact ethnic disparities in dementia and are important to consider when planning policy. This criterion additionally meant that we excluded papers not differentiating between medical and social services, or institutionalizations in nursing home versus hospital. See Supplementary Box S2 for additional discussion on social care.

We also excluded studies on differences in diagnosis rates/delays. Delayed diagnoses of dementia may influence time to presentation or use of other services as well, although relationships between different points on the pathway to care are not explored here.

This review only included articles comparing differences in service use between different ethnicity groups. However, studies describing service use patterns in individual ME groups will also provide valuable insights for policy and planning.

Many of the included studies recruited participants from AD centers and memory clinics or used data from clinic records or electronic health records. These sampling methods may limit bias from nonresponse and loss‐to‐follow‐up. However, these designs fail to capture individuals who do not access services at all and may overestimate service use in ME and comparison groups, particularly if recruitment processes differ between ME groups at research‐focused AD centers. Given the literature on barriers to accessing memory services, participants recruited from memory services may not be representative of all individuals with dementia.

Study quality in this review was mixed, and ethnicity was defined/reported in unstandardized ways (Supplementary Box S1). For example, some U.S. studies used “White” categories which included Hispanic White individuals, whereas others specified non‐Hispanic White groups. In the UK, “White British” groups were sometimes combined with “other White” groups. Ethnicity was often not self‐ascertained.

Finally, our secondary objective of examining differences in cognition/severity at presentation was included because we wanted to compare this metric, thought to indicate delays to accessing services, to literature directly measuring time delays to presentation. We felt that our search strategy was likely to capture these papers, but because this was not explicitly defined as an objective in our initial protocol, our methodology was not specifically designed around this.

## CONCLUSION

There was little evidence of differences or delays in use of memory services in any ME group in any country, though studies may not have been sufficiently powered. ME groups may present to these services with higher cognitive impairment scores, although it is unclear whether this is due to delaying care. Primary care was not widely studied and should be considered in future research.

There was strong evidence that Black and African American groups in the U.S. are more frequently admitted to inpatient hospital services versus comparison groups. This finding was not reported in other ME groups in the U.S. In the UK, White British groups had higher inpatient admissions than other ME groups in one study. Further research is needed on emergency services use.

More research on service use in ME groups outside the U.S. is necessary. Increased accuracy and consistency in reporting ethnicity will improve our understanding of these findings.

## Supporting information


**Supplementary Table S1**. Summary of studies included by country. This table includes a summary of the number of studies in each country studying each race/ethnicity group and service.
**Supplementary Table S2**. Study characteristics. This table includes details of characteristics for each study including sample size and demographics of participants.
**Supplementary Table S3**. NOS results for cross‐sectional studies. This table includes additional details of the Newcastle‐Ottawa Scale assessment results for cross‐sectional studies and outcomes, broken down by individual NOS items.
**Supplementary Table S4**. NOS results for cohort studies. This table includes additional details of the Newcastle‐Ottawa Scale assessment results for cohort studies, broken down by individual NOS items.
**Supplementary Table S5**. Detailed U.S. service use results. This table gives further details of the results and statistics from U.S. studies on service use.
**Supplementary Table S6**. Detailed service use results from other countries. This table gives further details of the results and statistics from studies on service use in Belgium, the Netherlands, and the UK.
**Supplementary Table S7**. Detailed severity/cognition at presentation results from other countries. This table gives further details of the results and statistics from studies on severity or cognition at presentation to services in all countries which included this outcome.
**Supplementary Table S8**. Summary of results in U.S. studies by ethnicity and outcome. This shows a tabulation of U.S. studies by ethnicity groups and by type of health service examined.
**Supplementary Box S1**. Interpretation of race/ethnicity in this review. This box further details how we defined, searched for, and use ethnicity terms in this review.
**Supplementary Box S2**. Further context on social and long‐term care. This box places the findings of our review into context with findings from social services such as long‐term care.
**Supplementary Appendix S1**. Protocol submitted to PROSPERO.
**Supplementary Appendix S2**. PRISMA checklist.
**Supplementary Appendix S3**. Search strategy and list of search terms used.
