# Supplementary material for: Access to Health Services in Older Minority Ethnic Groups with Dementia: A Systematic Review
Source: J Am Geriatr Soc. 2020 Nov 24;69(3):822–34. doi: 10.1111/jgs.16929 (PMC7984264; doi:10.1111/jgs.16929)
Supplement: Supplementary file 1 — Supplementary Table S1. Summary of studies included by country. This table includes a summary of the number of studies in each country studying each race/ethnicity group and service. Supplementary Table S2. Study characteristics. This table includes details of characteristics for each study including sample size and demographics of participants. Supplementary Table S3. NOS results for cross‐sectional studies. This table includes additional details of the Newcastle‐Ottawa Scale assessment results for cross‐sectional studies and outcomes, broken down by individual NOS items. Supplementary Table S4. NOS results for cohort studies. This table includes additional details of the Newcastle‐Ottawa Scale assessment results for cohort studies, broken down by individual NOS items. Supplementary Table S5. Detailed U.S. service use results. This table gives further details of the results and statistics from U.S. studies on service use. Supplementary Table S6. Detailed service use results from other countries. This table gives further details of the results and statistics from studies on service use in Belgium, the Netherlands, and the UK. Supplementary Table S7. Detailed severity/cognition at presentation results from other countries. This table gives further details of the results and statistics from studies on severity or cognition at presentation to services in all countries which included this outcome. Supplementary Table S8. Summary of results in U.S. studies by ethnicity and outcome. This shows a tabulation of U.S. studies by ethnicity groups and by type of health service examined. Supplementary Box S1. Interpretation of race/ethnicity in this review. This box further details how we defined, searched for, and use ethnicity terms in this review. Supplementary Box S2. Further context on social and long‐term care. This box places the findings of our review into context with findings from social services such as long‐term care. Supplementary Appendix S1. Protocol submitted to [file JGS-69-822-s001.pdf]

Supplementary Table S1: Summary of included studies, by country

Ethnicity	Number of studies, n	Service	Number of studies, n
USA	13		
White/ Non-Hispanic White	13	Routine services	7
Black/African American	9	Primary care	1
Hispanic/Latino	4	Memory services/ AD centre	5
Native American	1	Outpatient (unspecified)	2
Asian American*	2	Acute care services	8
Chinese	1	Emergency department	1
Filipino	1	Inpatient hospital	6
Korean	1	ICU	1
Other/Non-White/not specified	2	Psychiatric	1
UK	4		
Black Caribbean/Black African	3	Routine services	2
East/South Asian	1	Memory clinic	2
Mixed/Other/not specified	3	Acute care services	2
White British	4	Emergency	1
Other White	2	Inpatient	1
Belgium	1		
Belgian-born	1	Memory clinic	1
European immigrants	1		
Non-European immigrants	1		
Australia	1		
English speaking background (ESB)	1	Memory service	1
Non-English speaking background (NESB)	1		
Netherlands	1		

Dutch	1	Inpatient readmission	1
Indonesian	1		
Turkish	1		
Surinamese	1		
Antillean	1		

*Our grouping. Studies looked at specific Asian American groups, which is why this category is broken down further

Supplementary Table S2: Study demographics

Study	Study years	Total n	Ethnicities (% of total sample)	% female	Mean age (at baseline if longitudinal)
**LoGiudice et al (2001)**	~2001 (not stated)	354	English speaking background (ESB) (77.1%)<br>non-English speaking background (NESB) (22.9%)	ESB: 65.2%, NESB: 56.8%	ESB: 76.2 (7.9), NESB: 71.2 (8.3)
**Segers et al (2013)**	2005-2012	380	Belgian-born (81.4%)<br>European immigrants (10.0%)<br>non-European immigrants (8.6%)	Belgian-born: 69.3%<br>European immigrants: 70.0%<br>Non-European immigrants: 54.2%	Belgian-born: female: 80.7, male: 78.2<br>European: female: 79.2, male: 78.8<br>Non-European: female: 71.5, male: 73.6
**Agyemang et al (2017)**	2000-2010	55,827	Dutch (96.7%)<br>Indonesian (2.4%)<br>Turkish (0.2%)<br>Surinamese (0.6%)<br>Antillean (0.1%)<br>(based on place of birth)	61.0%	81.5
**Knapp et al (2016)**	2006-2012	3,075	(1) Caribbean, African, or other Black (10.1%)<br>(2) East Asian or South Asian (3.1%)<br>(3) Mixed, unknown, and other (4.6%)<br>(4) White British or Other White (82.2%)	67.0%	34% between 70-79 years, 48.3% between 80-89 years
**Park et al (2017)**	2014-2015	1,420	White/White British (94.5%)<br>Other ethnicity (5.5%)	52.3%	77.9 (8.5)
**Sleeman et al (2018)**	2008-2013	4,867	White British (71.9%)<br>Other White (10.6%)<br>African Caribbean (8.8%)<br>Other (3.9%)<br>Not known (4.8%)	61.3%	85.3 (at death)
**Tuerk & Sauer (2015)**	2011-2012	239	White British (65.7%)<br>Black and minority ethnic (BME, including only Caribbean and African) (34.3%)	not stated	White British: 80.2 (8.8)<br>BME: 77.2 (7.1)
**Akpaffiong et al (1999)**	1993-1997	197	Caucasian (73.1%)<br>African American (26.9%)	2.03%	Caucasian: 73.6, African American: 75.3

**Chow et al (2000)**	1993-1995	7,000 (922 at follow-up)	Caucasian (94.4%)<br>Filipino (0.9%)<br>Asian (includes Chinese, Japanese, Korean) (4.7%)	Asian: 51.4%, Filipino: 61.5%, Caucasian: 54.4%	Asian: 70.3 (9.7), Filipino 72.1 (8.8), Caucasian 71.6 (8.9)
**Cohen & Carlin (1993)**	~1993 (not stated)	170	White (60%)<br>Black (40%)	75.0%	74.2
**Cox (1996)**	~1996 (not stated)	179	African American (55.3%)<br>White (44.7%)	African American: 60.6%, White: 86.3%	African American: 80.2, White: 83.9
**Gaugler et al (2006)**	1989-1994	8,125	Latino (4%) Caucasian (87.4%)<br>African American (8.6%)	60.3%	Latino: 78.3, Caucasian: 79.0, African American: 78.7
**Gessert et al (2006)**	2000-2002	3,170	White (83.4%)<br>Non-white (16.6%)	76.4%	87.3
**Husaini et al (2003)**	2008	5,556	White (87.2%)<br>Black (12.8%)	87.3%	82
**Husaini et al (2015)**	1991-1993	1,366	White (86.8%)<br>African American (13.2%)	70.5%	75 (full sample, including some without dementia)
**Livney et al (2011)**	1989-2008	1,128	African American (18.0%)<br>Hispanic/Latino (13.7%)<br>White Non-Hispanic (68.3%)	Overall 64.8%<br>African American: 73.4%, Hispanic/Latino: 63%, White Non-Hispanic: 62.9%,	Overall 75.3 (SD 8.4)<br>African American: 76.6 (7.4), Hispanic/Latino: 71.3 (9.7), White Non-Hispanic 75.7 (8.2)
**Miller et al (2009)**	2001-2004	421	Non-Hispanic White (79%) Other (21%)	55.8%	77.9 (7.5)
**Ornstein et al (2018)**	1999-2010	86	non-Hispanic White (19.9%)<br>non-Hispanic Black (25.3%)<br>Hispanic (54.8%)	67.44%	85.6 (6.7)
**Watari & Gatz (2004)**	1992-1997	272	Korean American (22%)<br>African American (16%)<br>Latino/a (29%)<br>European American (33%)	Korean American: 68%, African American: 77%, Latino/a: 71%, European American: 55%	Korean American: 76.1 (10.0), African American: 78.0 (8.2), Latino/a American: 75.0 (9.6)<br>European American: 78.7 (8.9)
**Weiner et al (2003)**	1993-2002	599	Native American (15%)<br>White (85%)	66.1%	Age at onset: 69.9, Age at evaluation: 73.6

Supplementary Table S3: NOS results for cross-sectional studies

Study	Representativ-ness	Sample size	Non-respondents	Ascertainment of exposure	Comparability	Assessment of the outcome	Statistical test	Ethnicity as the main predictor (yes or no)	Ethnicity reported in results (yes or no)
**Akpaffiong et al (1999)**			*+			**		yes	yes
**Chow et al (2000)**	*		*+	*		**		yes	yes
**Cohen & Carlin (1993)**	*		*+	*				yes	yes
**Cox (1996)**						*		yes	yes
**Gaugler et al (2006)**							*	yes	yes
**Gessert et al (2006)**			*+		*	**	*	no	yes
**Husaini et al (2003)**			*+			**		yes	yes
**Husaini et al (2015)**	*		*+			**		yes	yes
**Livney et al (2011)**	*			*	**		*	yes	yes
**LoGiudice et al (2001)** cognitive assessment / use of services	*		*+			* / **	/ *	yes	yes
**Park et al (2017)**	*				**	**	*	no	yes
**Segers et al (2013)** cognitive assessment / diagnostic delay	*		*+		** /	** / *	/ *	yes	yes
**Tuerk & Sauer (2015)**	*		*+			**	*	yes	yes
**Watari & Gatz (2004)** cognitive assessments / delay to seeking help and services used	*	*	*+			** / *	/	yes	yes
**Weiner et al (2003)**	*		*+	*			*	yes	yes

Items with a / indicate that there were at least two outcomes examined which differed in rating; the total was calculated by taking the average in each box.

Scoring:

- Representativeness: * for representative sample
- Sample size: * for justification of sample size (e.g. satisfactory power calculation for the outcome)
- Non-respondents: * for comparability between respondents and non-respondents (non-respondents do not differ on ethnicity), *+ where data was collected from routine data, no star if not discussed.
- Ascertainment of exposure: * for self-report
- Comparability: * controls for age, sex. ** controls for additional sociodemographic variables such as education or socioeconomic status or clinical variables such as severity, comorbidities, functional impairment, etc.
- Assessment of the outcome: ** for record linkage or clinician rating for severity/cognition outcomes, * for self-report, no star for no description
- Statistical test: * appropriate statistical test, measurement of association presented, including confidence intervals and p-value, no star if no description, missing details, or not appropriate.

Supplementary Table S4: NOS results for cohort studies

Study	Representa-tiveness of the exposed cohort	Selection of the non-exposed cohort	Ascertain-ment of exposure	Demonstra-tion that the outcome of interest was not present at the start of the study	Comparabil-ity of cohorts on the basis of the design or analysis	Assessment of outcome	Was follow-up long enough for outcomes to occur	Adequacy of follow up of cohorts	Ethnicity as the main predictor variable (yes or no)	Ethnicity reported in results (yes or no)
**Agyemang et al (2017)**	*	*		*+	**	*	*	*+	yes	yes
**Chow et al (2000)**	*	*	*	*			*		yes	yes
**Knapp et al (2016)**	*	*		*	**	*		*+	no	yes
**Miller et al (2009)**		*			**				no	yes
**Ornstein et al (2018)**	*	*	*	*		*	*		yes	yes
**Sleeman et al (2018)**	*	*		n/a	**	*	*	*+	no	yes

Scoring:

- Representativeness of the exposed cohort: * for representativeness
- Selection of the non-exposed cohort: * if used same population pool for all racial/ethnic groups
- Ascertainment of exposure: * for self-report
- Demonstration that the outcome of interest was not present at the start of the study: * for first presentation to services or first readmission
- Comparability of cohorts on the basis of the design or analysis: * controls for age, sex. ** controls for additional sociodemographic variables such as education or socioeconomic status or clinical variables such as severity, comorbidities, functional impairment, etc.
- Assessment of outcome: * for record linkage or independent blind assessment, no star for other or not mentioned
- Was follow-up long enough for outcomes to occur: * for >=1 years of follow-up, no star if shorter or not mentioned in article
- Adequacy of follow up of cohorts: * for complete follow-up or <20% loss plus description, no star if loss to follow-up was associated with race/ethnicity, *+ if not mentioned but study came from routine health record data.

Supplementary Table S5: Detailed USA service use results

Study	Outcome definition	Type of statistic	Statistic value and significance	Covariates	Adjusted statistic	Key findings
Chow et al (2000)	Frequency of use of primary medical care before baseline, frequency of referral and use of outpatient primary medical care referrals.	Mean, % (frequency)	Frequency of use of primary medical care prior to baseline (n): Asian 0.83 (46), Filipino 1 (12), Pacific Islander 0.5 (2), Caucasian 0.88 (864)<br><br>Frequency of referrals to primary medical care: Asian 0.96, Filipino 1.00, Caucasian 0.95<br><br>Frequency of referrals completed (obtained service) to primary medical care: Asian 0.95, Filipino 0.92, Caucasian 0.95			Significant differences between different race/ethnic groups for other referrals and services (e.g. social), but not primary medical care. Based on proportions in the population, they concluded that Asian and Pacific Islander groups under-enrol at AD centres, but this was not included because uncertainty of prevalence of AD in those populations.
Cohen & Carlin (1993)	Years of having symptoms prior to presenting to assessment centre, having a prior evaluation before presenting to centre	Mean, % (frequency)	Mean years of symptoms prior to presentation:<br>Black 3.9, White 3.5, t= 0.87, p=0.383<br><br>% with prior evaluation to presentation: Black: 67.3%, White 71.2%, X^2 = 0.23, p=0.630			No race/ethnic differences in time before accessing the service, and no difference in accessing prior evaluation.
Cox (1996)	Number of hospitalisations in last 12 months, number of hospital days	Mean	Mean number hospitalisations: African American: 2.55, White: 1.96, t=-2.10, p<0.05<br><br>Mean number of hospital days: African American: 15.32, White: 11.41, t=-1.36, p>0.05			African American individuals with dementia had more hospitalisations in the 12 months prior to the study.
Gaugler et al (2006)	Number of overnight hospital stays in the past 6 months	Mean (SD)	Latino: 3.97 (10.62), Caucasian: 8.96 (0.11), African American: 11.10 (0.42)			The African American group's mean number of overnight hospital stays was higher than the Caucasian group's mean number of overnight hospital

						stays in the past six months (11.1 compared to 8.96).
Gessert et al (2006)	Use of hospital or intensive care unit (ICU) in last 90 days of life	Odds ratio (OR)	OR of Nonwhite group compared to White reference, stratified by rural vs urban nursing home setting.<br><br>Hospitalisation: Rural: 1.54 (1.03-2.32) p=0.04, Urban: 2.41 (1.83-3.18) p<0.001<br><br>Hospitalisation >10 days: Rural: 1.27 (0.75-2.16) p=0.38, Urban 2.45 (1.74-3.45) p<0.001<br><br>ICU: Rural 1.31 (0.68-2.53) p=0.41, Urban 1.59 (1.12-2.25) p=0.01			Nonwhite ethnicity was associated with higher use of hospitalisation and ICU in final 90 days of life as compared to the White reference group, particularly in urban setting.
Husaini et al (2003)	Additional admissions to hospital, number of hospital days	Mean	Additional admissions: Black: 2.60, White: 2.46, p<0.001<br><br>Hospital days: Black: 21.4, White: 16.7, p<0.001			There was a higher number of re-admissions and higher number of days spent in hospital in the Black race/ethnic group as compared to the White race/ethnic group.
Husaini et al (2015)	Average number of inpatient days, average number of outpatient visits, average number of physician visits, % of group who had visited emergency services	Mean, %	Mean inpatient days: White 7.89, African American 12.42, p<0.05 (SD and exact value not reported)<br><br>Mean outpatient visits: White: 3.17, African American 2.88, p>0.05<br><br>Mean physician visits: White: 24.15, African American 26.22, p>0.05			African American people with dementia had more inpatient hospital days than white people with dementia, but outpatient visits, physician visits, and emergency services use did not differ between the two groups.

			% using emergency services:<br>White: 68.9%, African American 75.0%, p>0.05			
Livney et al (2011)	Time between onset and initial presentation to services	Mean (SD), Beta coefficient		Age, sex, years of education	Time between onset and initial presentation to services<br>African American: 3.1 (2.12), Latino 3.3 (3.32), White Non-Hispanic 3.4 (2.29), overall p=0.15<br>Comparing African American to White Non-Hispanic: B= -0.38, p=0.05<br>Comparing Latino to White Non-Hispanic: B= -0.08, p=0.76<br>Comparing African American to Latino: B= -0.30, p=0.30	No significant differences between the time from onset to first presentation at the AD Centre.
Miller et al (2009)	Use of service in the preceding month at baseline, 3, 6, and 9 months after assignment to treatment or placebo	X^2p-value, OR (95% c.i.)	Inpatient hospital: p=0.11<br>Any outpatient: p=0.0018<br>AD-related outpatient: p=0.012<br>Mental-health outpatient: p=0.001<br>Medical-surgical outpatient: p=0.81	Age, gender, marital status, education, AD Cooperative Study-ADL, Neuropsychiatric Inventory, MMSE, AD-Related Quality of Life, Health Utilities Index, time from baseline	With Other as reference, OR (95% c.i.) of Non-Hispanic White participants' use:<br>Inpatient hospital: 0.81 (0.43-1.51)<br>Any outpatient: 1.67 (1.10-2.52)<br>AD-related outpatient: 1.53 (1.00-2.35)<br>Mental health outpatient: 0.85 (0.51-1.40)<br>Medical-surgical outpatient: 1.08 (0.77-1.52)	Non-Hispanic White trial participants were more likely to use any outpatient services in the last month than other participants after adjusting for other factors, particularly AD-related outpatient services. However, differences in odds of inpatient hospital use, mental health outpatient use, and medical-surgical outpatient use disappeared after adjustment.<br>The per-month average across the whole sample of service use was: 71.1% using outpatient services, 44% using AD-specific services, and 4.5% using inpatient services.

Ornstein et al (2018)	Mean number of hospital admissions, hospital days, ICU days, and hospice days from diagnosis to death	Mean (SD)	Hospital admissions, *p<0.05: Total 5.22(5.55), non-Hispanic White 3.58 (4.53), non-Hispanic Black 7.09 (6.92)*, Hispanic 5.22 (5.01)<br>Hospital days:<br>Total 72.78 (88.45), non-Hispanic White 61.00 (78.71), non-Hispanic Black 104.43 (95.33)*, Hispanic 61.38 (87.98)<br>ICU days:<br>Total 3.27 (6.05), non-Hispanic White 2.73 (4.80), non-Hispanic Black 4.35 (6.34), Hispanic 2.97 (6.70)			Non-Hispanic Black individuals with dementia had almost two times the hospital admissions compared to the non-Hispanic White group, and the fewest hospice days among all groups. They also had the most hospital and ICU days. There were no statistical differences between Hispanic and non-Hispanic White groups.
Watari & Gatz (2004)	Years to seeking help, mean number of services used	Mean (SD), p-values from ANOVA/ANCOVA	Years to seek help:<br>Korean American: 3.27 (2.68), African American: 3.85 (3.00), Latino/a: 4.35 (3.45), European American: 3.78 (3.10), p>0.05 comparing Korean American to all other groups combined or between all four groups<br>Mean number of services used: Korean American .60, non-Korean American .86, F=2.89, p <0.05	Years to seek help: ANCOVA, controlling for education and income	Years to seek help: no significant difference	No differences between ethnicities in delay before attendance or number of services used prior to clinic attendance.
Weiner et al (2003)	Time from onset of symptoms to initial evaluation	Mean (Standard Error)	Time to evaluation: Native American: 4.01 (0.35), White: 3.74 (0.11), t=-0.87, p=0.39			No differences between Native American and White groups for time to evaluation from onset.

Abbreviations: AD- Alzheimer's Disease, CI- confidence interval, HR- hazard ratio IRR- incidence rate ratio, ICU- Intensive Care Unit, ME- minority ethnic, MMSE- Mini-Mental State Examination, OR- odds ratio, SD- standard deviation

Supplementary Table S6: Detailed service use study results from Belgium, the Netherlands, and the UK

Study	Country	Outcome definition	Type of statistic	Statistic value and significance	Covariates	Adjusted statistic	Key findings
Segers et al (2013)	Belgium	Carer-estimated time between first symptoms and first consultation	Mean, SD	Delay to presentation (years):<br>Belgian-born: 1.9 (1.8), European: 1.5 (0.8), Non-European: 3.2 (3.1), p>0.05			No statistically significant differences between migrant groups and Belgian-born groups for delay in presenting to memory services.
Agyemang et al (2017)	Netherlands	Risk of readmission after first hospitalisation or day clinic visit for dementia	Hazard ratio (HR)	Inpatient:<br>Ethnic Dutch 1.00<br>Indonesian: 1.00 (0.90–1.12)<br>Surinamese: 1.25 (1.01–1.53)*<br>Turkish: 1.85 (1.27–2.69)*<br>Antillean: 1.21 (0.73–2.01)<br><br>Day clinic:<br>Ethnic Dutch: 1.00<br>Indonesian: 0.95 (0.83–1.07)<br>Surinamese: 0.98 (0.76–1.25)<br>Turkish: 0.74 (0.47–1.17)<br>Antillean: 0.96 (0.58–1.59)<br><br>*p<0.05	Age, sex, comorbidity	Model 2, Inpatient:<br>Ethnic Dutch: 1.00<br>Indonesian: 0.97 (0.87–1.07)<br>Surinamese: 1.15 (0.94–1.42)<br>Turkish: 1.41 (0.96–2.05)<br>Antillean: 1.18 (0.71–1.95)<br><br>Model 2, Day clinic:<br>Ethnic Dutch: 1.00<br>Indonesian: 0.95 (0.84–1.08)<br>Surinamese: 0.98 (0.76–1.25)<br>Turkish: 0.70 (0.45–1.10)<br>Antillean: 0.90 (0.54–1.50)	Higher unadjusted risk of readmission for inpatients who are Surinamese or Turkish as compared to Dutch. After adjustment for age, sex, and comorbidities, there were no differences in readmission risk between race/ethnic groups.
Knapp et al (2016)	UK	Odds of being admitted in a 6-month period after a MMSE assessment (note unit of measurement was 6-month periods, so some patients double-counted)	Odds ratio (OR)		MMSE, Year of MMSE, age, inpatient general or mental health admission in previous 12 months, gender, Health of the Nations Outcome Scales variables	With White British/White Other as a reference group:<br>OR of general inpatient admission in 6 months:<br>Caribbean/African: 0.68 (0.53-0.88), p <0.01<br>East/South Asian: 0.43 (0.25-0.73), p<0.01<br>Mixed/Unknown: 1.35 (0.93-1.96), p=0.11<br><br>OR of mental health inpatient admission in 6 months:<br>Caribbean/African: 0.89 (0.54-	The Caribbean/African and East/South Asian groups both had lower odds of being admitted to general inpatient wards as compared to the White British/White Other groups.

						1.47), p=0.64<br>East/South Asian: 1.21 (0.53-2.75), p=0.66<br>Mixed/Unknown: 0.86 (0.36-2.09), p=0.75	
Sleeman et al (2018)	UK	Number of emergency department attendances in last year before death	Incidence Rate Ratio (IRR)	Unadjusted not reported, but authors state African Caribbean ethnicity vs. White British ethnicity was associated with more emergency department attendances.	Gender, age at death, index of multiple deprivation, primary diagnosis, Health of the Nations Outcome Scale, MMSE score, time since last mental health care contact, care home residence, year of death	IRR of emergency department attendance in last year of life:<br>White British: reference<br>Other White: 0.99 (0.89-1.09), p=0.78<br>African Caribbean: 1.07 (0.95-1.19), p = 0.26<br>Other: 1.08 (0.92-1.27), p=0.33<br>Not known: 1.19 (0.84-1.69), p=0.33	As compared to White British groups, ME groups did not have different rates of emergency department attendance in the last year of life after controlling for other variables. African Caribbean individuals with dementia had higher rates of emergency department attendance as compared to White British individuals with dementia before adjustment. On average, all groups had 2.1 attendances (SD 2.3, range 0–54). 73.1-87.2% of participants in each ethnicity group had at least one admission.

Abbreviations: HR- hazard ratio, IRR- incidence rate ratio, ME- minority ethnic, MMSE- Mini-Mental State Examination, OR- odds ratio, SD- standard deviation

Supplementary Table S7: Detailed severity/cognition at presentation study results (all countries)

Study	Outcome definition	Type of statistic	Statistic value and significance	Covariates	Adjusted statistic	Key findings
**Australia**						
LoGiudice et al (2001)	CDR at presentation, MMSE at presentation	Mean (SD), X^2	MMSE:<br>ESB: 18.0 (5.3), NESB: 14.7 (6.2), p<0.001<br><br>CDR:<br>X^2= 14.3, p=0.003<br><br>CAMCOG:<br>ESB: 58.2 (17.0), NESB: 49.2 (20.8)			People from NESB were more likely to present with more severe cognitive impairment and at later stages of dementia based on MMSE, CAMCOG, and CDR.
**Belgium**						
Segers et al (2013)	MMSE at first presentation	Mean (SD)	MMSE:<br>Belgian-born: 22.2 (4.6), European 19.5 (6.2) p<0.05, Non-European 14.0 (6.4) p<0.0001	Sex, migration status, age >79, education, vascular lesions	OR for having MMSE <21 of immigrant compared to Belgian-born: 6.5	Non-European immigrants had lower MMSE scores than Belgian-born and European immigrants even after controlling for education.
**United Kingdom**						
Park et al (2017)	Cognitive function tertile at first referral based on MMSE score or other cognitive test.	Odds ratio	OR for Other ethnicity (not White/White British) compared to White/White British: 1.3, using clinic as cluster	Age, sex, deprivation (IMD), number of comorbidities, clinic	OR for Other ethnicity (not White/White British) compared to White/White British: 1.3 (1.1-1.7), p<0.05	Individuals not in the White/White British ethnicity group were more likely to present to memory assessment services with lower cognitive function scores.
Tuerk & Sauer (2015)	ACE-R score and MMSE score at presentation/ diagnosis	Mean (SD)	ACE-R: White British: 57.4 (13.5), BME: 48.7 (11.2), p<0.001<br>MMSE: White British: 21.0 (4.6), BME: 20.1 (4.1), p=0.20			BME patients scored lower on the ACE-R as compared to White British patients at first presentation to memory service; MMSE scores were not significantly different.
**United States**						

Akpaffiong et al (1999)	MMSE score at admission	Mean (SD)	Mean (SD):<br>Caucasian: 15.6 (8.3), African American 14.7 (7.5)<br>t-test: t = 0.69 p = 0.49 (calculated from data given in paper)			No significant differences in MMSE at admission between different ethnic groups. Length of hospital stay was also reported but not tested (34 days in Caucasian group and 32 in African American group).
Chow et al (2000)	MMSE at baseline evaluation	Mean (SD)	Mean MMSE (SD):<br>Asian 15.4 (7.1), Filipino 15.1 (7.6), Pacific Islander 17.5 (6.1), Caucasian 17.7 (7.3), p<0.01 for Asian vs Caucasian and Filipino vs Caucasian.			Lower mean MMSE at baseline for Filipino and Asian groups compared to Caucasian group.
Livney et al (2011)	MMSE at presentation, Global cognition index at presentation, CDR (Clinical Dementia Rating) at presentation, DSRS (Dementia Severity Rating Scale) at presentation	Mean (SD), Beta coefficient		Age, sex, years of education	MMSE at presentation<br>African American: 17.6 (5.16), Latino 15.1 (5.91), White Non-Hispanic 20.7 (5.34), overall p<0.0001<br>Comparing African American to White Non-Hispanic: B=-1.78, p<0.0001<br>Comparing Latino to White Non-Hispanic: B=-1.50, p=0.0074<br>Comparing African American to Latino: B=-0.284, p=0.64	Adjusting for education attenuated or greatly reduced many race/ethnicity differences in cognition score at presentation, although African American individuals and Latino individuals with AD still had significantly lower scores than White Non-Hispanic individuals on the MMSE and Global Cognition Index. Severity (based on DSRS and CDR) at presentation was higher in the African American group as compared to the White Non-Hispanic group, and higher in the Latino group as compared to the African American group for CDR.
Watari & Gatz (2004)	Severity at presentation based on BDRS-CERAD and MMSE	Mean (SD), p-values from ANOVA/ ANCOVA		MMSE and BDRS-CERAD: ANCOVA, controlling for education	BDRS-CERAD:<br>Korean American: 4.16 (4.00), African American: 5.66 (4.03), Latino/a: 4.75 (4.69), European American: 3.86 (4.33), p>0.05<br>MMSE:<br>Korean American: 16.03 (7.62), African American: 13.56 (7.35), Latino/a: 16.35 (7.95), European American: 18.89 (7.99), F=3.21, p<0.05	Lower MMSE scores in African American group compared to White/European American group. No differences in BDRS-CERAD.

					Scheffé post hoc analysis: African American group mean score was significantly lower than White/European American group mean score, F=2.64, p<0.05	
Weiner et al (2003)	MMSE score at initial evaluation	Mean (Standard Error)	MMSE: Native American: 17.74 (0.78), White: 18.48 (0.27), t=0.98, p=0.33			No differences between Native American and White groups in MMSE at evaluation.

Abbreviations: ACE-R- Addenbrooke's Cognitive Examination Revised, AD- Alzheimer's Disease, BDRS-CERAD- Blessed–Roth Dementia Scale Rating (Consortium to Establish a Registry for Alzheimer's Disease version), BME- Black and minority ethnic, CAMCOG- cognitive section of the Cambridge Examination for Mental Disorders of the Elderly, CDR-Clinical Dementia Rating, CI- confidence interval, DSRS- Dementia Severity Rating Scale, ESB- English speaking background, MMSE- Mini-Mental State Examination, NESB- Non-English speaking background, OR- odds ratio, SD- standard deviation

Supplementary Table S8: Summary of results from US studies by service and ethnicity

	Routine care services			*Acute care and crisis services*			*Cognition at presentation (MMSE)*	
	Primary care (n=1)	*Memory services-time to presentation (n=4)*	*Outpatient services (not differentiated by level of care) (n=2)*	*Hospital and ICU-number of days spent (n=4)*	*Hospital and ICU- number of inpatient stays (n=6)*	*Emergency services (n=1)*	*Memory services (n=4)*	*Psychiatric inpatient unit (n=1)*
African American or Black (n=9)		**2 no difference** (Cohen & Carlin, Watari & Gatz), **1 shorter time to presentation** (Livney et al.)	**1 no difference** (Husaini et al. 2015)	**1 no difference** (Cox), **3 higher number of inpatient days** (Ornstein et al., Husaini et al. 2003, Husaini et al. 2015)	**4 higher number of admissions** (Cox, Gaugler et al., Husaini et al. 2003, Ornstein et al.)	**1 no difference in use** (Husaini et al. 2015)	**2 lower** (Livney et al., Watari & Gatz)	**1 no difference** (Akpaffiong et al.)
Asian American (n=1)	**1 no difference** (Chow et al.)						**1 lower** (Chow et al.)	
Filipino (n=1)	**1 no difference** (Chow et al.)						**1 lower** (Chow et al.)	
Hispanic or Latino (n=4)		**2 no difference** (Livney et al., Watari & Gatz)		**1 no difference in hospital/ ICU days** (Ornstein et al)	**2 no difference in inpatient admissions** (Gaugler et al., Ornstein et al.)		**1 lower** (Livney et al.) **1 no difference** (Watari & Gatz)	
Korean American (n=1)		**1 no difference** (Watari & Gatz)					**1 no difference** (Watari & Gatz)	
Native American/ American Indian (n=1)		**1 no difference** (Weiner et al.)					**1 no difference** (Weiner et al.)	
White, European American, Caucasian, Non-Hispanic White compared to other ethnicities (n=2)			**1 higher use of any outpatient compared to other ethnicity groups** (Miller et al.)		**1 lower number of hospital stays in last 90 days of life compared to non-White** (Gessert et al.), **1 no difference in inpatient use** (Miller et al.)			

This table summarises results in US studies by ethnicity (versus comparison groups) and service. MMSE results were reported for severity/cognition because it was the most frequently used.

Supplementary Box 1: Interpretation of race/ethnicity in this review

Defining race and ethnicity:

Our interpretation of ethnicity in this review is informed by Bhopal's definition that "ethnicity is a multi-faceted quality that refers to the group to which people belong, and/or are perceived to belong, as a result of certain shared characteristics, including geographical and ancestral origins, but particularly cultural traditions and languages."[1] While "race" conceptually differs from "ethnicity", particularly in its historical implications, both are socially constructed and often used interchangeably such that for the purposes of our review we used either the hybrid "race/ethnicity" or "ethnicity" alone.[1]

Race/ethnicity terms in our search strategy:

In our search terms, we included race/ethnicity categorisations from national censuses in addition to "race" and "ethnicity". We expected many studies to rely on these as standard (albeit imperfect) groupings, particularly where ethnicity was gathered from routine data sources.[1] In the US census, race is grouped into five census categories: White, Black or African American, Asian, American Indian or Alaska Native, Native Hawaiian and Pacific Islander, and other.[2] "Hispanic origin" is considered as an ethnicity and separate question in the census,[3] but for the purposes of research are often integrated with race categories. In the UK, suggested census categories from the Office of National Statistics are: White, Mixed/Multiple ethnic groups, Asian/Asian British, Black/ African/Caribbean/Black British, and Other ethnic group.[4]

Consistency of terminology to describe race/ethnicity categories in our review:

While we maintained the authors' chosen terms when discussing individual studies in order to reflect the specific category used (which could affect how participants self-ascribed), many of the broader terms used may hide important disparities within groups. For example, the use of terms such as "White", "European", and "Caucasian" has been criticised because they are imprecise, can imply geographic or even genetic (rather than social) divisions, and can refer to heterogeneous populations with different health burdens.[5]

Supplementary Box 2: Further context: Social and long-term care services

Long-term residential care services, such as nursing homes, comprise an important source of care as well for people living with dementia. While they are distinct from the other routine and acute medical services described in this review due to the additional social services included, disparities in use of these services have also been reported and may also impact use of services included in this review.

Previous reviews and meta-analyses have found that ME groups such as African American and Hispanic American groups were less likely to use long-term or nursing home facilities versus comparison groups.[6-8] In qualitative studies from the UK, South Asian families and carers have also expressed negative views about using residential care services and a strong preference for being cared for at home.[9] These patterns of nursing and residential care services may then impact use of the services included in this review, for example, if nursing homes or residential care services are better able to reduce preventable hospitalisations and manage dementia outside the hospital.[10] Within nursing home facilities, however, there have also been reports of ethnic disparities in dementia care management.[11]

Future research might examine the disparities in use of social services, including updating existing reviews on the topic, as well as investigating how use of social services influences the use of the hospital, memory clinic, and primary care services studied in our review.

Systematic review

1. * Review title.

Give the working title of the review, for example the one used for obtaining funding. Ideally the title should state succinctly the interventions or exposures being reviewed and the associated health or social problems. Where appropriate, the title should use the PI(E)COS structure to contain information on the Participants, Intervention (or Exposure) and Comparison groups, the Outcomes to be measured and Study designs to be included.

Differences in rate of health care service utilization in ethnic minorities with dementia: a systematic review

2. Original language title.

For reviews in languages other than English, this field should be used to enter the title in the language of the review. This will be displayed together with the English language title.

3. * Anticipated or actual start date.

Give the date when the systematic review commenced, or is expected to commence.

07/11/2018

4. * Anticipated completion date.

Give the date by which the review is expected to be completed.

31/12/2019

5. * Stage of review at time of this submission.

Indicate the stage of progress of the review by ticking the relevant Started and Completed boxes. Additional information may be added in the free text box provided.
Please note: Reviews that have progressed beyond the point of completing data extraction at the time of initial registration are not eligible for inclusion in PROSPERO. Should evidence of incorrect status and/or completion date being supplied at the time of submission come to light, the content of the PROSPERO record will be removed leaving only the title and named contact details and a statement that inaccuracies in the stage of the review date had been identified.
This field should be updated when any amendments are made to a published record and on completion and publication of the review. If this field was pre-populated from the initial screening questions then you are not able to edit it until the record is published.

The review has not yet started: No

Review stage	Started	Completed
Preliminary searches	Yes	Yes
Piloting of the study selection process	Yes	Yes
Formal screening of search results against eligibility criteria	Yes	No
Data extraction	No	No
Risk of bias (quality) assessment	No	No
Data analysis	No	No

Provide any other relevant information about the stage of the review here (e.g. Funded proposal, protocol not yet finalised).

Search was performed on 7 November 2018. Title and abstract screening is ongoing at the time of this submission.

Search was performed on 7 November 2018. Title and abstract screening is ongoing at the time of this submission.

6. * Named contact.

The named contact acts as the guarantor for the accuracy of the information presented in the register record.

Melissa Co

Email salutation (e.g. "Dr Smith" or "Joanne") for correspondence:

Ms Co

7. * Named contact email.

Give the electronic mail address of the named contact.

melissa.co@kcl.ac.uk

8. Named contact address

Give the full postal address for the named contact.

Health Service and Population Research Department Institute of Psychiatry, Psychology & Neuroscience De Crespigny Park London SE5 8AF

9. Named contact phone number.

Give the telephone number for the named contact, including international dialling code.

10. * Organisational affiliation of the review.

Full title of the organisational affiliations for this review and website address if available. This field may be completed as 'None' if the review is not affiliated to any organisation.

Health Service and Population Research Department, Institute of Psychiatry, Psychology & Neuroscience, King's College London

Organisation web address:

https://www.kcl.ac.uk/ioppn/depts/hspr/index.aspx

11. * Review team members and their organisational affiliations.

Give the personal details and the organisational affiliations of each member of the review team. Affiliation refers to groups or organisations to which review team members belong. **NOTE: email and country are now mandatory fields for each person.**

Ms Melissa Co. King's College London

12. * Funding sources/sponsors.

Give details of the individuals, organizations, groups or other legal entities who take responsibility for initiating, managing, sponsoring and/or financing the review. Include any unique identification numbers assigned to the review by the individuals or bodies listed.

This review is part of a PhD funded by King's College London Centre for Doctoral Studies.

Grant number(s)

13. * Conflicts of interest.

List any conditions that could lead to actual or perceived undue influence on judgements concerning the main topic investigated in the review.

None

14. Collaborators.

Give the name and affiliation of any individuals or organisations who are working on the review but who are not listed as review team members. **NOTE: email and country are now mandatory fields for each person.**

15. * Review question.

State the question(s) to be addressed by the review, clearly and precisely. Review questions may be specific or broad. It may be appropriate to break very broad questions down into a series of related more specific questions. Questions may be framed or refined using PI(E)COS where relevant.

To determine if ethnic group differences in rates of contact with health services (e.g. inpatient admission or outpatient visit with general medical care, psychiatric services, memory clinics, or emergency services) have been quantified in the literature for individuals with dementia.

16. * Searches.

State the sources that will be searched. Give the search dates, and any restrictions (e.g. language or publication period). Do NOT enter the full search strategy (it may be provided as a link or attachment.)

The search will be conducted in Ovid and will include English-language, peer reviewed articles from: Embase (1974 to 2018 Week 45), Ovid MEDLINE (1946 to November 6, 2018), Global Health (1973 to 2018 Week 43), and PsycINFO (1806 to October Week 4). The search strategy will be shared with protocol 118129 "Differences in mortality rates in ethnic minorities with dementia: a systematic review".

17. URL to search strategy.

Give a link to a published pdf/word document detailing either the search strategy or an example of a search strategy for a specific database if available (including the keywords that will be used in the search strategies), or upload your search strategy.Do NOT provide links to your search results.

https://www.crd.york.ac.uk/PROSPEROFILES/118132_STRATEGY_20181128.pdf

Alternatively, upload your search strategy to CRD in pdf format. Please note that by doing so you are consenting to the file being made publicly accessible.

Do not make this file publicly available until the review is complete

18. * Condition or domain being studied.

Give a short description of the disease, condition or healthcare domain being studied. This could include health and wellbeing outcomes.

Dementia (all causes), healthcare utilization.

19. * Participants/population.

Give summary criteria for the participants or populations being studied by the review. The preferred format includes details of both inclusion and exclusion criteria.

Individuals with a diagnosis of dementia, of different ethnic groups, of any age. This can include vascular dementia (whose incidence is associated with ethnicity) as well as mild cognitive impairments (MCI). Studies focusing on services for caregivers of people with dementia will be excluded. Studies defining their population based on other disorders will be excluded (e.g. studies whose population is defined to be people with cancer, some of whom may/may not have dementia). Studies which also include participants with other diseases, entire hospital registers, or more general populations may be included only if they separately report results for a dementia-specific group (e.g. studies which include both participants with cancer and participants with dementia but separately report the results for those with dementia). Studies of dementia diagnosis in a general population (since the population does not have a dementia diagnosis at the start of the study) will be excluded.

20. * Intervention(s), exposure(s).

Give full and clear descriptions or definitions of the nature of the interventions or the exposures to be reviewed.

Ethnicity; Ethnic minority individuals with dementia including MCI and vascular dementia.

21. * Comparator(s)/control.

Where relevant, give details of the alternatives against which the main subject/topic of the review will be compared (e.g. another intervention or a non-exposed control group). The preferred format includes details of both inclusion and exclusion criteria.

Ethnic majority individuals (e.g. White British in the UK) with dementia. Studies which do not compare multiple ethnic groups will be excluded. Studies comparing multiple ethnic minorities will also be included.

22. * Types of study to be included.

Give details of the types of study (study designs) eligible for inclusion in the review. If there are no restrictions on the types of study design eligible for inclusion, or certain study types are excluded, this should be stated. The preferred format includes details of both inclusion and exclusion criteria.

Full-text articles in peer-reviewed journals with quantitative, observational study designs. Interventional studies (e.g. implementation) and trials will be excluded. Studies must have used ethnicity as a predictor variable, even if it is not the main hypothesis of the study.

23. Context.

Give summary details of the setting and other relevant characteristics which help define the inclusion or exclusion criteria.

24. * Main outcome(s).

Give the pre-specified main (most important) outcomes of the review, including details of how the outcome is defined and measured and when these measurement are made, if these are part of the review inclusion criteria.

Use of healthcare services: This is defined to include inpatient admission or outpatient visit with general medical care, psychiatric services, memory clinics, or emergency services. This may include studies on referrals, use of services, presentation to services at different rates, etc. These can be dementia-specific services or general services, but not services specific to other unrelated conditions (e.g. studies looking at how dementia patients access cancer services would be excluded). This does not include nursing and residential homes, which are considered social services, or diagnostic services with general practitioners/primary care providers. Studies about willingness to use services or advanced care directives will not be included, as they represent attitudes towards health services rather than use of services (as defined above).

* Measures of effect

Please specify the effect measure(s) for you main outcome(s) e.g. relative risks, odds ratios, risk difference, and/or 'number needed to treat.

Service use must occur for a population which already has dementia (i.e. not diagnostic).

25. * Additional outcome(s).

List the pre-specified additional outcomes of the review, with a similar level of detail to that required for main outcomes. Where there are no additional outcomes please state ‘None’ or ‘Not applicable’ as appropriate to the review

None

* Measures of effect

Please specify the effect measure(s) for you additional outcome(s) e.g. relative risks, odds ratios, risk difference, and/or 'number needed to treat.

Not applicable.

26. * Data extraction (selection and coding).

Describe how studies will be selected for inclusion. State what data will be extracted or obtained. State how this will be done and recorded.

All hits for the search will be de-duplicated in Ovid and exported to EndNote, where further duplicates will be removed. Then, the remaining papers will be imported to Rayyan (rayyan.qcri.org, Ouzzani 2016), a web application which facilitates screening and collaboration for systematic reviews. The review author will first screen all records for inclusion, and a secondary reviewer will screen a percentage of the records. Relevant records after title and abstract screening will then be assessed for eligibility after reading the full-text article. The secondary reviewer will also assess a percentage of the relevant records for eligibility. Disagreements between the two reviewers will be discussed and brought to a third reviewer for a final decision, and the review author will contact authors of articles in question if more information on the study design or measures is necessary. As the search strategy is being shared with another review (protocol 118129), screening for inclusion in either will be done concurrently. Additional articles may also be included in the results after manually reviewing reference lists of included articles from the search. Results from the screen and reasons for exclusion after full-text screening will be presented in a PRISMA flow diagram.

Data will be extracted by the review author into a standard Excel form which will include information about authors, funding source, publication date, setting/service, study design, recruitment method, inclusion/exclusion criteria, sample size, type of dementia diagnosis, ethnic groupings, other variables included in analyses, and health service use findings.

27. * Risk of bias (quality) assessment.

Describe the method of assessing risk of bias or quality assessment. State which characteristics of the studies will be assessed and any formal risk of bias tools that will be used.

Quality of studies and risk of bias will be assessed by the primary researcher using the Newcastle-Ottawa Scale (http://www.ohri.ca/programs/clinical_epidemiology/oxford.asp). The checklist will also be modified to include an item identifying whether ethnicity was the primary exposure being investigated or a secondary exposure variable. Studies will be given an overall quality rating of high, moderate, low, or unclear (not enough information reported).

28. * Strategy for data synthesis.

Provide details of the planned synthesis including a rationale for the methods selected. This **must not be generic text** but should be **specific to your review** and describe how the proposed analysis will be applied to your data.

It is expected that there will be multiple health service types and multiple study designs included in the review, so narrative synthesis will be used to summarize findings from these articles. Number of studies discussing each health service type as well as how often disparities were found between ethnic groups and in what direction will be reported. Quality of the studies will also be taken into consideration.

29. * Analysis of subgroups or subsets.

State any planned investigation of ‘subgroups’. Be clear and specific about which type of study or participant will be included in each group or covariate investigated. State the planned analytic approach.

This is a narrative synthesis as it is expected that there will be many types of health service utilization outcomes reported; thus, it is not possible to specify subgroup analyses in advance.

30. * Type and method of review.

Select the type of review and the review method from the lists below. Select the health area(s) of interest for your review.

Type of review

Cost effectiveness
No

Diagnostic
No

Epidemiologic
No

Individual patient data (IPD) meta-analysis
No

Intervention
No

Meta-analysis
No

Methodology
No

Narrative synthesis
Yes

Network meta-analysis
No

Pre-clinical
No

Prevention
No

Prognostic
No

Prospective meta-analysis (PMA)
No

Review of reviews
No

Service delivery
No

Synthesis of qualitative studies
No

Systematic review
Yes

Other
No

Health area of the review

Alcohol/substance misuse/abuse
No

Blood and immune system

No

Cancer
No

Cardiovascular
No

Care of the elderly
No

Child health
No

Complementary therapies
No

COVID-19
No

Crime and justice
No

Dental
No

Digestive system
No

Ear, nose and throat
No

Education
No

Endocrine and metabolic disorders
No

Eye disorders
No

General interest
No

Genetics
No

Health inequalities/health equity
Yes

Infections and infestations
No

International development
No

Mental health and behavioural conditions
Yes

Musculoskeletal
No

Neurological
Yes

Nursing
No

Obstetrics and gynaecology
No

Oral health
No

Palliative care
No

Perioperative care
No

Physiotherapy
No

Pregnancy and childbirth
No

Public health (including social determinants of health)
Yes

Rehabilitation
No

Respiratory disorders
No

Service delivery
Yes

Skin disorders
No

Social care
Yes

Surgery
No

Tropical Medicine
No

Urological
No

Wounds, injuries and accidents
No

Violence and abuse
No

31. Language.

Select each language individually to add it to the list below, use the bin icon to remove any added in error.
English

There is not an English language summary

32. * Country.

Select the country in which the review is being carried out from the drop down list. For multi-national collaborations select all the countries involved.

England

33. Other registration details.

Give the name of any organisation where the systematic review title or protocol is registered (such as with The Campbell Collaboration, or The Joanna Briggs Institute) together with any unique identification number assigned. (N.B. Registration details for Cochrane protocols will be automatically entered). If extracted data will be stored and made available through a repository such as the Systematic Review Data Repository (SRDR), details and a link should be included here. If none, leave blank.

34. Reference and/or URL for published protocol.

Give the citation and link for the published protocol, if there is one

Give the link to the published protocol.

Alternatively, upload your published protocol to CRD in pdf format. Please note that by doing so you are consenting to the file being made publicly accessible.

No I do not make this file publicly available until the review is complete

Please note that the information required in the PROSPERO registration form must be completed in full even if access to a protocol is given.

35. Dissemination plans.

Give brief details of plans for communicating essential messages from the review to the appropriate audiences.

This review is expected to be submitted for publication in a peer-reviewed journal and also included as part of a PhD thesis at King's College London.

Do you intend to publish the review on completion?

Yes

36. Keywords.

Give words or phrases that best describe the review. Separate keywords with a semicolon or new line. Keywords will help users find the review in the Register (the words do not appear in the public record but are included in searches). Be as specific and precise as possible. Avoid acronyms and abbreviations unless these are in wide use.

dementia

Alzheimer's Disease

ethnicity

race

service use

service utilization

access to health services

37. Details of any existing review of the same topic by the same authors.

Give details of earlier versions of the systematic review if an update of an existing review is being registered, including full bibliographic reference if possible.

38. * Current review status.

Review status should be updated when the review is completed and when it is published. For newregistrations the review must be Ongoing.
Please provide anticipated publication date

Review_Ongoing

39. Any additional information.

Provide any other information the review team feel is relevant to the registration of the review.

40. Details of final report/publication(s) or preprints if available.

This field should be left empty until details of the completed review are available OR you have a link to a

preprint.

Give the link to the published review.

Supplementary Appendix S2: PRISMA checklist

Section/topic	#	Checklist item	Reported on page #
**TITLE**			
Title	1	Identify the report as a systematic review, meta-analysis, or both.	1, 2
**ABSTRACT**			
Structured summary	2	Provide a structured summary including, as applicable: background; objectives; data sources; study eligibility criteria, participants, and interventions; study appraisal and synthesis methods; results; limitations; conclusions and implications of key findings; systematic review registration number.	2
**INTRODUCTION**			
Rationale	3	Describe the rationale for the review in the context of what is already known.	5
Objectives	4	Provide an explicit statement of questions being addressed with reference to participants, interventions, comparisons, outcomes, and study design (PICOS).	5
**METHODS**			
Protocol and registration	5	Indicate if a review protocol exists, if and where it can be accessed (e.g., Web address), and, if available, provide registration information including registration number.	5
Eligibility criteria	6	Specify study characteristics (e.g., PICOS, length of follow-up) and report characteristics (e.g., years considered, language, publication status) used as criteria for eligibility, giving rationale.	5
Information sources	7	Describe all information sources (e.g., databases with dates of coverage, contact with study authors to identify additional studies) in the search and date last searched.	6
Search	8	Present full electronic search strategy for at least one database, including any limits used, such that it could be repeated.	Appendix 1,3
Study selection	9	State the process for selecting studies (i.e., screening, eligibility, included in systematic review, and, if applicable, included in the meta-analysis).	6
Data collection process	10	Describe method of data extraction from reports (e.g., piloted forms, independently, in duplicate) and any processes for obtaining and confirming data from investigators.	6
Data items	11	List and define all variables for which data were sought (e.g., PICOS, funding sources) and any assumptions and simplifications made.	6-7
Risk of bias in individual studies	12	Describe methods used for assessing risk of bias of individual studies (including specification of whether this was done at the study or outcome level), and how this information is to be used in any data synthesis.	7
Summary measures	13	State the principal summary measures (e.g., risk ratio, difference in means).	6-7
Synthesis of results	14	Describe the methods of handling data and combining results of studies, if done, including measures of consistency (e.g., I^2) for each meta-analysis.	7

Section/topic	#	Checklist item	Reported on page #
Risk of bias across studies	15	Specify any assessment of risk of bias that may affect the cumulative evidence (e.g., publication bias, selective reporting within studies).	7
Additional analyses	16	Describe methods of additional analyses (e.g., sensitivity or subgroup analyses, meta-regression), if done, indicating which were pre-specified.	7
**RESULTS**			
Study selection	17	Give numbers of studies screened, assessed for eligibility, and included in the review, with reasons for exclusions at each stage, ideally with a flow diagram.	8, 26
Study characteristics	18	For each study, present characteristics for which data were extracted (e.g., study size, PICOS, follow-up period) and provide the citations.	27, Table S2
Risk of bias within studies	19	Present data on risk of bias of each study and, if available, any outcome level assessment (see item 12).	29, Table S3 & S4
Results of individual studies	20	For all outcomes considered (benefits or harms), present, for each study: (a) simple summary data for each intervention group (b) effect estimates and confidence intervals, ideally with a forest plot.	30-32, Table S8
Synthesis of results	21	Present results of each meta-analysis done, including confidence intervals and measures of consistency.	9-12 (no meta-analysis)
Risk of bias across studies	22	Present results of any assessment of risk of bias across studies (see Item 15).	N/A
Additional analysis	23	Give results of additional analyses, if done (e.g., sensitivity or subgroup analyses, meta-regression [see Item 16]).	12
**DISCUSSION**			
Summary of evidence	24	Summarize the main findings including the strength of evidence for each main outcome; consider their relevance to key groups (e.g., healthcare providers, users, and policy makers).	12-14
Limitations	25	Discuss limitations at study and outcome level (e.g., risk of bias), and at review-level (e.g., incomplete retrieval of identified research, reporting bias).	15-16
Conclusions	26	Provide a general interpretation of the results in the context of other evidence, and implications for future research.	17
**FUNDING**			
Funding	27	Describe sources of funding for the systematic review and other support (e.g., supply of data); role of funders for the systematic review.	18

From: Moher D, Liberati A, Tetzlaff J, Altman DG, The PRISMA Group (2009). Preferred Reporting Items for Systematic Reviews and Meta-Analyses: The PRISMA Statement. PLoS Med 6(7): e1000097. doi:10.1371/journal.pmed1000097

For more information, visit: **www.prisma-statement.org**.

Supplementary Appendix S3: Search strategy

***Note:** This review is part of a larger project investigating ethnic disparities in mortality and service use in dementia and shares a search strategy with another review focusing on mortality (PROSPERO: CRD42018118129). Thus, search terms for survival and mortality are also included in the search strategy. Screening for both reviews was done concurrently.*

The search strategy will be developed using Ovid. Broadly, the search will follow this structure: dementia search terms AND ethnicity search terms AND (Mortality search terms OR Service utilization search terms), with search terms listed below. Subject headings are indicated by (SH) following the term.

Dementia search terms combined with OR: dementia (SH), Alzheimer disease (SH), dement*, Alzheimer*

Ethnicity search terms combined with OR: ethnicity (SH), race (SH), minority group (SH), ethnic group (SH), ethnic group (SH), ethnic*, B?ME, race, racial, minorit*, multi ethnic*, multi?ethnic*, multi?racial*, multi racial, multi cultur*, multi?cultur*, Asian*, Black*, African*, Hispanic*, Latin*, Caucasian*, White*, East Asian (SH), Southeast Asian (SH), Asian American (SH), British Asian (SH), Asian (SH), South Asian (SH), West Asian (SH), Central Asian (SH), African American (SH), African Caribbean (SH), West African (SH), African (SH), South African (SH), African Brazilian (SH), Southern African (SH), East African (SH), North African (SH), Hispanic (SH), Caucasian (SH)

Mortality search terms combined with OR: hospital mortality (SH), mortality risk (SH), mortality (SH), mortality rate (SH), mortality, survival (SH), survival analysis (SH), surviv*

Service use search terms combined with OR: hospital (SH), health service (SH), mental health service (SH), emergency health service (SH), service use, primary care, primary medical care (SH), GP, general practi*, accident and emergenc*, ER, emergency, hospitalis*, hospitaliz*, hospital utilization (SH), memory clinic

Ovid databases search:

1 exp dementia/

2 dement*

3 exp Alzheimer disease/

4 Alzheimer*

5 1 or 2 or 3 or 4

6 exp ethnic group/ or exp ethnicity/ or exp race/

7 ethnic*

8 B?ME

9 exp minority group/ or exp ethnic group/

10 race

11 racial

12 minorit*

13 multi ethnic*

14 multi?ethnic*

15 multi?racial

16 multi racial

17 multi cultur*

18 multi?cultur*

19 exp East Asian/ or exp Southeast Asian/ or exp Asian American/ or exp British Asian/ or exp Asian/ or exp South Asian/ or exp West Asian/ or exp Central Asian/

20 Asian*

21 exp African American/ or exp African Caribbean/ or exp West African/ or exp African/ or exp South African/ or exp African Brazilian/ or exp Southern African/ or exp East African/ or exp North African/

22 black*

23 African*

24 exp Hispanic/

25 hispanic*

26 latin*

27 exp Caucasian/

28 caucasian*

29 white*

30 6 or 7 or 8 or 9 or 10 or 11 or 12 or 13 or 14 or 15 or 16 or 17 or 18 or 19 or 20 or 21 or 22 or 23 or 24 or 25 or 26 or 27 or 28 or 29

31 exp hospital mortality/ or exp mortality risk/ or exp mortality/ or exp mortality rate/

32 mortality

33 exp survival/ or exp survival analysis/

34 surviv*

35 31 or 32 or 33 or 34

36 exp hospital/ or exp health service/ or exp mental health service/ or exp emergency health service/

37	"service use"
38	"primary care"
39	exp primary medical care/
40	GP
41	"general practi*"
42	"accident and emergenc*"
43	ER
44	emergency
45	hospitalis*
46	hospitaliz*
47	exp hospital utilization/
48	"memory clinic"
49	5 and 30 and 35
50	36 or 37 or 38 or 39 or 40 or 41 or 42 or 43 or 44 or 45 or 46 or 47 or 48
51	35 or 40
52	5 and 30 and 50

References for supplementary material

[1] Bhopal R. Glossary of terms relating to ethnicity and race: for reflection and debate. *Journal of Epidemiology & Community Health*. 2004;**58**: 441-445.

[2] U.S. Census Bureau. 2020 Census Questions: Race. 2020.

[3] Grieco EM, Cassidy RC. Overview of Race and Hispanic Origin. In: U.S. Census Bureau, ed., 2001.

[4] Office of National Statistics. Ethnic group, national identity and religion. *Measuring equality: A guide for the collection and classification of ethnic group, national identity and religion data in the UK*, 2016.

[5] Bhopal R, Donaldson L. White, European, Western, Caucasian, or what? Inappropriate labeling in research on race, ethnicity, and health. *American journal of public health*. 1998;**88**: 1303-1307.

[6] Cooper C, Tandy AR, Balamurali TBS, Livingston G. A systematic review and meta-analysis of ethnic differences in use of dementia treatment, care, and research. *American Journal of Geriatric Psychiatry*. 2010;**18**: 193-203.

[7] Gaugler JE, Yu F, Krichbaum K, Wyman JF. Predictors of nursing home admission for persons with dementia. *Medical care*. 2009: 191-198.

[8] Luppa M, Luck T, Brähler E, König H-H, Riedel-Heller SG. Prediction of institutionalisation in dementia. *Dementia and geriatric cognitive disorders*. 2008;**26**: 65-78.

[9] Bowes A, Wilkinson H. ‘We didn’t know it would get that bad’: South Asian experiences of dementia and the service response. *Health & social care in the community*. 2003;**11**: 387-396.

[10] Feng Z, Coots LA, Kaganova Y, Wiener JM. Hospital and ED use among Medicare beneficiaries with dementia varies by setting and proximity to death. *Health affairs*. 2014;**33**: 683-690.

[11] Sengupta M, Decker SL, Harris-Kojetin L, Jones A. Racial differences in dementia care among nursing home residents. *Journal of aging and health*. 2012;**24**: 711-731.
